# Exploring the Impact of Student Teaching Apprenticeships on Student Achievement and Mentor Teachers

**DOI:** 10.1080/19345747.2019.1698087

**Published:** 2020-02-13

**Authors:** Dan Goldhaber, John M. Krieg, Roddy Theobald

**Affiliations:** a American Institutes for Research, Seattle, Washington, USA;; b University of Washington, Seattle, Washington, USA;; c Western Washington University, Bellingham, Washington, USA

**Keywords:** Value added, student teaching, long-term gains

## Abstract

We exploit within-teacher variation in the years that math and reading teachers in grades 4–8 host an apprentice (“student teacher”) in Washington State to estimate the causal effect of these apprenticeships on student achievement, both during the apprenticeship and afterwards. While the average causal effect of hosting a student teacher on student performance in the year of the apprenticeship is indistinguishable from zero in both math and reading, hosting a student teacher is found to have modest positive impacts on student math and reading achievement in a teacher’s classroom in following years. These findings suggest that schools and districts can participate in the student teaching process without fear of short-term decreases in student test scores while potentially gaining modest long-term test score increases.

## Introduction

Every year there are more than 125,000 student teachers who complete apprenticeships in K-12 public schools.^1^ These apprenticeships occur in the classrooms of (and are supervised by) inservice teachers known as mentor teachers (or “cooperating teachers” in Washington, the setting of this study). Does hosting these teacher candidates affect student test performance, either during the apprenticeship or in the classrooms of mentor teachers after they host a student teacher? As we describe below, there is a good deal of speculation about this, but no published quantitative exploration of the impacts on students in the classrooms where student teaching has taken place.^2^

The lack of information about how student teaching impacts K-12 students is problematic. States and localities make decisions about key aspects of student teaching that influence whether there are positive or negative effects on students and the quality of the apprenticeship. While specific state-level requirements for mentor teachers are relatively rare, state laws occasionally mandate aspects of the field placements in which student teaching occurs,^3^ such as the diversity of the school in which student teaching occurs or the effectiveness or qualifications of the mentor teachers.^4^ Nevertheless, teacher education programs (TEPs) often have trouble finding student teacher placements for their candidates because of the perception that student teaching may be disruptive in ways that negatively impact students (St. John, Goldhaber, Krieg, & Theobald, 2018).

In this article we explore the effects of hosting student teachers in grade 4–8 math and ELA classrooms on the achievement of students in the host classroom and for future students of the mentor teacher. In particular, we utilize a unique, longitudinal database of student teachers from 15 TEPs that place student teachers in Washington State public schools to address three inter-related questions: (1) Does hosting a student teacher have an impact on student achievement in the classrooms in which student teaching occurs?; (2) Does hosting a student teacher have an impact on student achievement in the classrooms of mentor teachers in years after student teaching occurs?; and (3) Do these effects vary according to the prior effectiveness of mentor teachers?

Relying on within-mentor estimates, we find little evidence that hosting a student teacher impacts student achievement during the year of student teaching, at least in the grades and subjects we consider. Specifically, the average causal effect of hosting a student teacher on contemporaneous student performance is indistinguishable from zero in both math and reading. However, in subsequent years we find modest positive impacts on student math and reading achievement of having supervised a student teacher. Under our identification strategy, this estimate could be biased by patterns of student assignments after a teacher hosts a student teacher, but we find no evidence of nonrandom sorting of stronger classrooms to teachers who previously hosted a student teacher, and this relationship is not different between schools that appear to track students to different classrooms on the basis of prior performance and schools that do not. Another possible route of bias would occur if mentors who host student teachers are on a steeper growth path than other teachers, but we also do not observe these trends in the data. Together, this supports the argument that these apprenticeships have benefits for mentor teachers that persist into the future.

## Background

Student teaching is widely regarded as the capstone to a teacher candidate’s preparation experience (Anderson & Stillman, 2013). Not surprisingly then, a good deal of academic literature describes the role student teaching plays in the development of teacher candidates (Borko & Mayfield, 1995; Clarke, Triggs, & Nielsen, 2014; Ganser, 2002; Graham, 2006; Hoffman et al., 2015; Zeichner, 2009). More recently, researchers have investigated how the attributes of internship schools (Goldhaber, Krieg, & Theobald, 2017; Ronfeldt, 2012, 2015) and mentor teachers (Goldhaber, Krieg, & Theobald, 2018; Ronfeldt, Brockman, & Campbell, 2018) influence the later outcomes of student teachers who become public school teachers. Importantly for this study, both Goldhaber et al. (2018) and Ronfeldt et al. (2018) find that teachers tend to have higher value when the mentor teacher of their student teaching placement has higher value added, all else equal, though Goldhaber et al. (2018) document that this relationship decays somewhat after candidates enter the workforce.

There is only a small academic literature addressing the ways in which schools or classrooms that host student teachers might themselves be affected, though much of this could be classified as speculation (St. John et al., 2018). But there are clear ways in which the role mentor teachers play in the mentorship of student teachers could lead to short- or longer-run effects on student achievement, either because of changes in resources or teaching practices. In the short-run, for instance, student teachers bring internship schools additional human resources, which might allow for more adult attention to the individual needs of students and greater differentiation of instruction; student teachers also bring to schools more recently adopted practices taught in TEPs (Hurd, 2007).

There is also some suggestion that hosting a student teacher could confer benefits to mentor teachers. Kerry and Shelton Mayes (1995), for instance, argue that that the act of helping student teachers dissect their classroom practices cause mentor teachers to reflect on their own practices in ways that lead to self-improvement.^5^ Field and Philpott (2000) provide survey evidence supporting the hypothesis as “mentors often claimed that they were forced to re-evaluate current practice in light of rationalizing their work to student teachers.”^6^ There is also some evidence of this type of “peer learning” among inservice teachers (Jackson & Bruegmann, 2009; Papay, Taylor, Tyler, & Laski, 2016).^7^

On the other hand, hosting student teachers requires substantial time and resource commitments by mentor teachers which could divert attention from students, decreasing their achievement. Moreover, given the clear evidence of positive student achievement benefits associated with having teachers with greater experience (e.g., Ladd & Sorensen, 2017; Rivkin, Hanushek, & Kain, 2005; Rockoff, 2004), and the possibility that experienced mentor teachers turn their classrooms over to inexperienced student teachers, we might expect negative effects on test scores in classrooms hosting student teachers.

These concerns are borne out in a qualitative study based on interviews with individuals responsible for student teacher placements in several TEPs, districts, and schools in Washington, the setting of this study (St. John et al., 2018). Specifically, principals and administrators responsible for student teaching placements in schools and districts reported protecting low-performing schools from student teachers on the assumption that these placements could be disruptive in these settings. This study also illustrates stark differences in attitudes about the desirability of potential mentor teachers; while many placement coordinators and district administrators stressed the importance of finding highly-effective mentor teachers to support candidate development, some principals reported recruiting teachers with “stagnant” teaching practices to serve as a mentor teacher on the hypothesis that those teachers will benefit from serving as a mentor (St. John et al., 2018, p. 16).

In short, the sparse literature touching on how hosting student teachers impacts student achievement does not provide a clear theoretical direction about what we should expect; rather, it suggests that the effects of hosting likely depend on how student teachers are utilized. TEPs often provide guidelines on the length of internships and the hours teacher candidates are required to be in the classroom, but little systemic information is known regarding the actual time breakdown of mentor-mentee interactions.^8^ It is generally understood that the hours mentor teachers typically spend mentoring, the frequency with which teacher candidates observe the mentor teacher in instruction, and the time mentor teachers observe instruction by the teacher candidate all vary both within and across TEPs (Greenberg, Pomerance, & Walsh, 2011). In some cases, having a student teacher may be highly interactive with the mentor-mentee relationship akin to a co-teaching environment (e.g., Heck & Bacharach, 2016), whereas in other scenarios mentor teachers may simply “hand off” the classroom and the corresponding responsibilities to the teacher candidate. While this analysis cannot test these differences directly, we can address whether—on average, across these different models of student teaching—hosting a student teacher appears to impact concurrent and subsequent student performance in a mentor teacher’s classroom.

A related strand of literature that relates to this work has to do with how mentor teachers are selected. In Washington State, student teaching positions are governed both by state code and contractual arrangements between TEPs and school districts. Washington is one of a handful of states that provide guidance to TEPs about the nature of student teaching placements (National Council for Accreditation of Teacher Education, 2010), but even these guidelines are vague, stating that “field experiences provide opportunity to work in communities with populations dissimilar to the background of the candidate.” This is commonly interpreted by TEPs as a mandate to place student teachers in racially diverse schools. Field placement agreements, however, generally state that the TEP and district will make “cooperative arrangements” to determine student teaching assignments. Finally, we observe few differences in student teaching assignments by TEP; for instance many are one quarter long, some are one semester long. Most happen in the spring, though some are in the fall.

To our knowledge only two research papers have explored mentor selection in Washington. In a quantitative study of Washington State student teaching placements, Krieg, Goldhaber, and Theobald (in press) find that mentors who supervise student teachers have more experience, higher degree levels, and higher value added in math than those who do not supervise student teachers. In a companion qualitative study, St. John et al. (2018) find that TEPs and districts tend to rely more on social networks and personal connections than other factors (such as teacher effectiveness) in matching candidates to mentor teachers. As described below, our empirical models explicitly control for experience and implicitly control for differences between teachers who do and do not host student teachers through the inclusion of teacher fixed effects.

## Data and Summary Statistics

The data set we utilize combines student teaching data about teacher candidates from institutions participating in Washington State’s Teacher Education Learning Collaborative (TELC) with K–12 administrative data provided by Washington State’s Office of the Superintendent of Public Instruction (OSPI). During the years of this study, the TELC data observes student teaching placements from 15 of the state’s 21 college and university-based Washington State TEPs. This data includes when student teaching occurred, the schools in which teacher candidates completed their student teaching, and the mentor teachers that supervised these internships.^9^

Though many of the institutions in TELC provided student teaching data going back to the mid-2000s and, in one case, to the late 1990s, we focus on student teaching data from 2009–10 to 2014–2015 in this analysis for two reasons. First, nearly all TEPs provided complete data about their teacher candidates over this time period.^10^ Second, these years of data correspond with years in which student-level data from OSPI can be linked to teachers through the state’s CEDARS data system, introduced in the 2009–2010 school year.^11^ By connecting the student teaching data from TELC institutions to the student-level data from OSPI, we create a dataset that links student teachers to: the K-12 students they taught during their student teaching placements; the students of the mentor teachers both before and after hosting the student teacher; and the public schools in which student teaching occurred.^12^

Importantly, this dataset can be further linked to a number of additional variables about students and teachers. Specifically, the student-level data from OSPI includes annual standardized test scores in math and English Language Arts (ELA) and demographic/program participation data for all K-12 students in the state, while the OSPI personnel data include information on teachers’ years of teaching experience. We standardize student test scores by grade and year and use these as the dependent variable in the analytic models described in the next section, while the other variables are used as control variables in these regressions.

**Figure 1.**
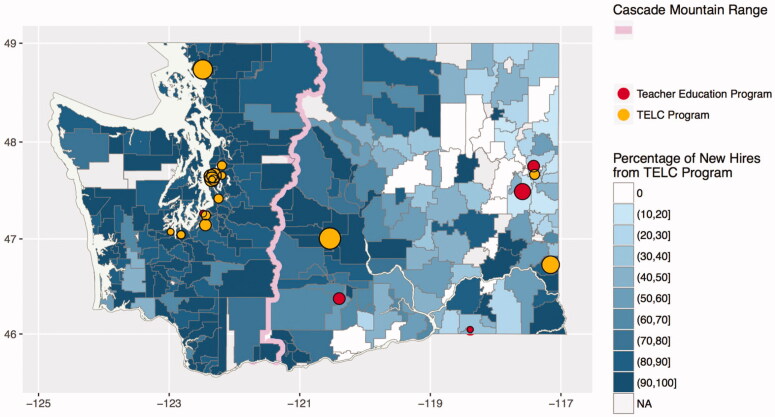
Percentage of newly-hired, in-state teachers from participating TEPs, 2010–2015. *Note.* TELC = Teacher Education Learning Collaborative (i.e., participating program); TEP = teacher education program. The diameter of the dot for each TEP is proportional to the number of newly-credentialed teachers from that TEP between 2010 and 2015.

As noted above, the TELC data include only 15 of the 21 TEPs that placed student teachers in Washington public schools during the 2009–2010 to 2014–2015 period. However, as shown in Figure 1, these participating TEPs are distributed unevenly throughout the state. Specifically, Figure 1 shows the percentage of newly-hired, in-state teachers between 2010 and 2015 in each district in the state who graduated from one of the TEPs participating in TELC (and thus included in this study).^13^ While 81 percent of all new teachers in Washington State are prepared by TEPs participating in TELC, this figure is 92% for districts west of the Cascade Mountains (the pink line in Figure 1) and just 55% for districts east of the Cascades.^14^ Because our empirical models rely on identifying which and when mentor teachers host student teachers, we include only observations west of the Cascades where we incorrectly mis-identify relatively few mentor teachers as not hosting student teachers.^15^

Since we focus on estimating the effects of hosting a student teacher on student test scores, we restrict the data to math and ELA teachers in grades 4–8 because students in these teachers’ classrooms can be linked both to current and prior test performance in these subjects. Based on these restrictions, our analytical sample includes 1,352 student teachers (1,106 unique mentor teachers) in math classrooms and 1,392 student teachers (1,128 unique mentor teachers) in ELA classrooms.^16^

Table 1 reports summary statistics for K-12 students who are in the classrooms of teachers who host a student teacher in at least one year of our observed data.^17^ We limit these summary statistics to teachers who host at least one student teacher because these are the teachers who identify the models as described in the next section, but these are not the only students and teachers who are included in our analytic models, as other students and teachers help identify the relationships between other control variables in our analytic models (e.g., prior student performance and teacher experience) and student achievement. Our complete analytic models observe 10,955 unique math and 11,453 unique ELA teachers who instructed about one million fourth through eighth graders.

**Table 1.** Student summary statistics (mentor teachers in math only).

	Before student teaching year	Student teaching year	After student teaching year
Current score in math	0.040	0.048	0.127***
	(0.970)	(0.967)	(1.004)
Current score in ELA	0.016	0.040	0.095***
	(0.933)	(0.935)	(0.950)
Lagged score in math	0.026	0.056	0.092*
	(0.976)	(0.992)	(0.995)
Lagged score in ELA	0.016	0.048	0.076*
	(0.965)	(0.974)	(0.968)
Female	0.491	0.491	0.493
American Indian	0.014	0.011**	0.009***
Asian / Pacific Isl.	0.119	0.130+	0.122
Black	0.059	0.068*	0.056
Hispanic	0.144	0.146	0.149
White	0.607	0.584*	0.594
Learning disability	0.057	0.056	0.054
Special Education	0.113	0.115	0.110
Gifted	0.061	0.069	0.074
Limited English	0.050	0.061***	0.061**
Free/Reduced Lunch	0.432	0.429	0.414
Unique teachers	1,106	1,106	1,106
Unique student teachers	0	1,352	0
Unique students	59,903	46,375	70,637

*Note.* ELA = English Language Arts. *p* Values from two-sided *t*-test relative to years before student teaching: +*p* < 0.10; *p < 0.05; **p < 0.01; ***p < 0.001.

Because the analysis described in the next section relies on within-teacher comparisons between years before, during, and after student teaching placements, we create indicators for these three different periods for each mentor teacher and present summary statistics separately for each period. In this and subsequent analyses, the “Before student teaching year” period is defined as all years before the *first* year a teacher hosted a student teacher; the “Student teaching year” (ST) period is defined as any years in which the teacher hosted a student teacher; and the “After student teaching year” (AF) period is defined as all years the teacher did not host a student teacher after the *first* year a teacher hosted a student teacher.

A few interesting findings from Table 1 are relevant for the analysis described in the next section. Most importantly, for both math and ELA, there is no difference in test performance among students in a teacher’s classroom before that teacher hosted a student teacher relative to the year when they serve as a mentor. However, student test scores are about one-tenth of a standard deviation above average for students of a teacher who hosted a student teacher in the past suggesting that teachers may gain from the experience of mentoring. An alternative explanation could be that mentors may receive better students after hosting a student teacher. We explore this with the lagged math and ELA scores in Table 1. These are the average student test scores from students the year prior to being in the mentor teacher’s classroom. The pattern among these students is similar to those of the current scores; mentors appear to have slightly better students in years after hosting a student teacher compared to those during and before hosting. On the other hand, by some measures, such as the proportion of students with Limited English Proficiency, it appears mentors have more disadvantaged classrooms in years following student teachers. This may reflect the possibility, discussed in St. John et al. (2018), that teachers are more likely to host a student teacher in years in which they have a more “difficult” classroom.

While we are able to control for these observable differences between the composition of the teacher’s classroom during the student teaching year and other years in the analytic models described in the next section, a key identifying assumption of these models is that teachers are no more or less likely to host a student teacher when they have a more difficult classroom along unobserved dimensions, conditional on the observed variables in Table 1. If this assumption is violated—e.g., if teachers are more likely to agree to host a student teacher when they have an “easier” classroom, or (conversely) seek out the help of a student teacher in years when they have a more difficult classroom—then these unobserved differences in classrooms will be attributed to the effect of hosting a student teacher. It is these concerns about unobserved differences in student assignment by mentor teacher status that motivate the robustness checks and falsification test that we describe in the next section.

## Analytic Approach

Our analytic approach follows Taylor and Tyler (2012), who rely on within-teacher variation in teacher evaluations to estimate the causal effect of these evaluations on concurrent and future student achievement. Our goal in this approach is to understand three related issues: (1) descriptively how teacher effectiveness (or “value added”) varies over time as a function of hosting a student teacher; (2) the causal impact of a student teacher on student tests scores during the student teaching year and years following; and (3) whether this impact differs based upon the mentor teacher’s own value added. Given these objectives, we use teacher “value added” models in three different ways: first (Equations 1–3), as a way to explore changes in teacher effectiveness over time; second (Equations 4 and 5), as a way to identify the causal impact of student teachers on students; and third (Equations 6–8), as a way to determine the *prior* effectiveness of mentor teachers which will then be used to explore any heterogeneity in the impact of student teachers by mentor teacher effectiveness.

The value-added estimates we use for our descriptive exploration are estimated from the following model:

(1) $$Y_{\mathrm{ijst}}=\alpha_{0}+\alpha_{1}Y_{i(t-1)}+\alpha_{2}S_{it}+\tau_{\mathrm{jst}}+\rho_{t}+\varepsilon_{\mathrm{ijst}}$$

The model in Equation 1 predicts student performance *Y_ijst_* for student *i* in the classroom of teacher *j* in subject *s* and year *t*, as a function of prior student performance, *Y_i(t−1)_*, additional student variables described in Table 1, *S_it_*, and a teacher-by-year fixed effect $\tau_{\mathrm{jst}}.$ To investigate changes in the annual value-added estimates ${\hat{\tau }}_{\mathrm{jst}}$ before, during, and after student teaching, let *t_j_’* be the first year that teacher *j* hosts a student teacher between 2010 and 2015 (defined only for teachers who host a student teacher). We then estimate the following second-stage model:

(2) $${\hat{\tau }}_{\mathrm{jst}}=\beta_{0}+\sum_{k=-3}^{3}\beta_{k+4}I\left(t-t_{j}^{\prime }=k\right)+\tau_{js}+\rho_{t}+\varepsilon_{\mathrm{jst}}$$

**Figure 2.**
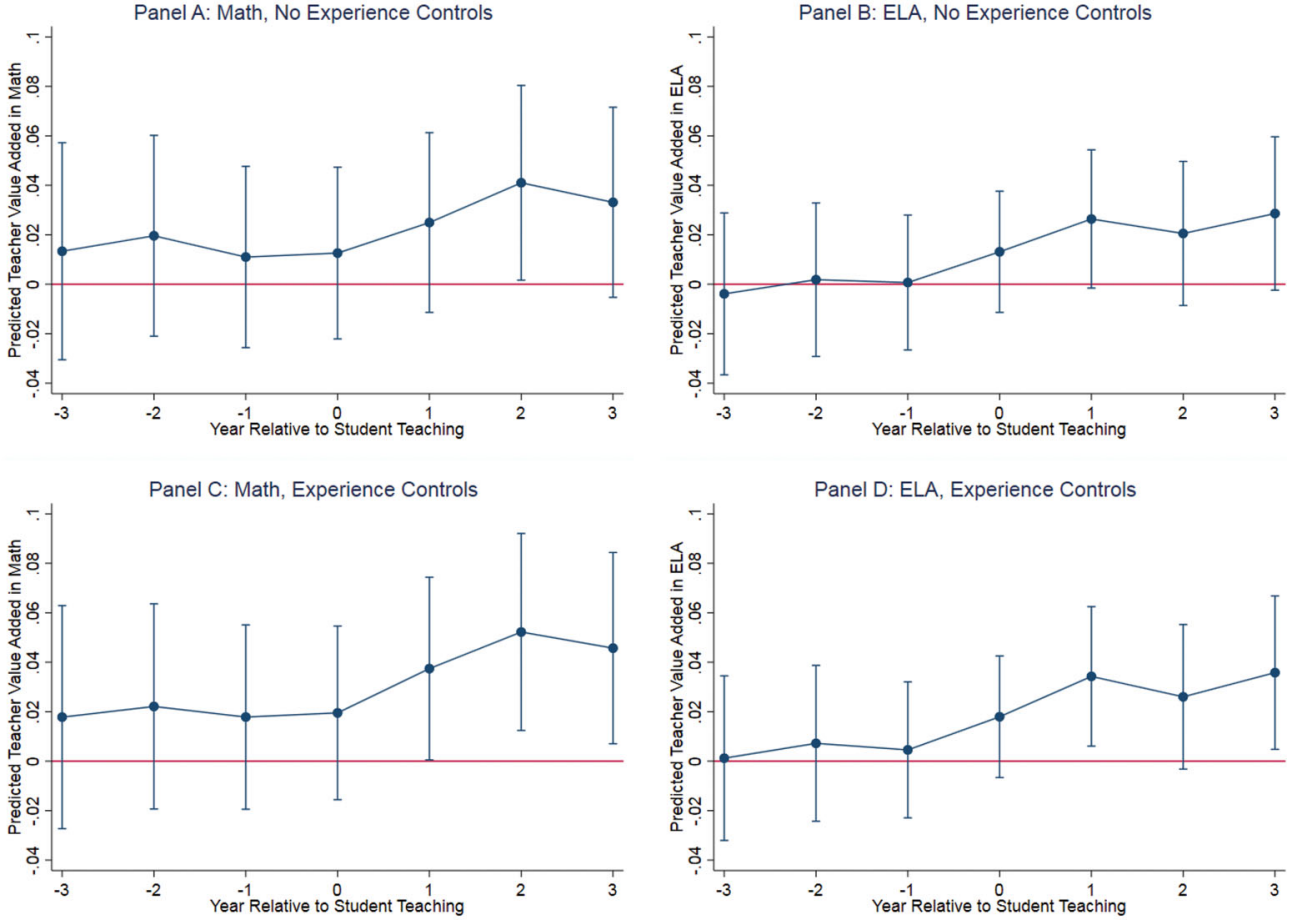
Average changes in value added for mentor teachers before, during, and after hosting a student teacher. *Note.* ELA = English Language Arts. Estimates and associated 95% confidence intervals from model predicting single-year teacher value added as a function of years relative to hosting a student teacher (year 0 is the student teaching year), a teacher fixed effect, and (in Panels C and D) indicators for teaching experience.

We estimate specifications of the model in Equation 2 that do and do not control for teacher experience—i.e., that do and do not account for average trends in effectiveness as teachers gain experience—and plot predicted value added from these models for different values of *k* in Figure 2. Because of the fixed effect $\tau_{js}$ in Equation 2, these estimates can be interpreted as average *within-teacher* trends in value added in the years before, during, and after hosting a student teacher.

We draw three preliminary conclusions from Figure 2 that inform the analytic approach outlined in the rest of this section. First, the fact that there is no visible trend in value added prior to teachers hosting a student teacher suggests that nonrandom selection on prior trends in teacher value added (e.g., teachers being selected to host a student teacher after a particularly “good year”) is not a large concern. Second, there is some suggestive evidence in Figure 2 that teacher value added may increase in the years *after* hosting a student teacher, which motivates the models described below. Finally, there is little evidence that experience controls meaningfully change trends in value added, likely because most mentor teachers are in the stage of their careers in which the relationship between experience and value added is relatively weak (e.g., Rockoff, 2004).

With that said, the returns to experience in Equation 2 (and the primary analytic models described below) are estimated using all teacher observations, not just those who served as a mentor. If mentor teachers have different returns to experience than other teachers—e.g., if teachers are selected to host a student teacher *because* of their rapid improvement as teachers—then the aggregated experience controls in our models will underestimate the effect of experience and misattribute it to the student teaching year and the years after student teaching. To check for this possibility, we create a binary variable *EverHost_j_* identifying whether teacher *j* ever hosted a student teacher between 2010 and 2015. We then use this variable to explore whether teachers who ever host a student teacher have differential returns to teaching experience:

(3) $${\hat{\tau }}_{\mathrm{jst}}=\alpha_{0}+\alpha_{1}{\mathrm{EverHost}}_{j}+\sum_{k}\beta_{k}\left({\mathrm{Exp}}_{\mathrm{jt}}\right)+\sum_{k}\delta_{k}\left({\mathrm{Exp}}_{\mathrm{jt}}\times {\mathrm{EverHost}}_{j}\right)+\varepsilon_{\mathrm{jt}}$$

If teachers who ever host a student teacher have different returns to experience, the δ coefficients in Equation 3 will be non-zero. However, when we estimate this model, no single δ is statistically different than zero. This motivates the aggregate teacher experience controls that we use throughout the remainder of the analysis.

We now turn to our primary analytic models that, following Taylor and Tyler (2012), make two substantive changes to the descriptive models described above: (1) estimate the relationship between hosting a student teacher and student achievement directly in one stage; and (2) combine the years before and after hosting a student teacher to improve the power of the models (and to be consistent with Table 1). Specifically, we begin our analysis by estimating a model of the form:

(4) $$Y_{\mathrm{ijst}}=\gamma_{0}+\gamma_{1}Y_{i(t-1)}+\gamma_{2}S_{it}+\gamma_{3}{ST}_{jt}+\sum_{k}\gamma_{k}I_{k}\left(\;\text{Exp}\;\right)+\tau_{js}+\rho_{t}+\varepsilon_{\mathrm{ijst}}$$

The two differences between Equations 1 and 4 is that 4 includes binary indicators for each year of teacher experience, *Exp_jt_*, and also includes an indicator for whether teacher *j* hosted a student teacher in year *t*, *ST_jt_*.^18^ The parameter of interest in Equation 4 is γ_3_, which can be interpreted as the average difference in student performance, all else equal, between years in which a teacher hosted a student teacher and years in which the *same* teacher did not host a student teacher. We cluster all standard errors at the teacher level.

We argue that identifying the impact of student teaching based on within-teacher over-time variation is preferable to identification in the cross-section. Prior evidence from Washington (Krieg, Theobald, & Goldhaber, 2016) demonstrates that mentor teachers are likely to be more effective teachers than non-mentor teachers, though qualitative research also points to the possibility that some apprenticeship assignments are made to help give mentor teachers “a break” (St. John et al., 2018) and thus may not be based on the instructional quality of the mentor. Either way, an unobserved correlation between teacher effectiveness and assignment as a mentor teacher would lead to a biased finding in a cross-section analysis of the impact of hosting a student teacher.

The $\tau_{js}$ in Equation 4 represents the baseline component of value added (due to the experience controls in the model) and account for time-invariant differences between teachers; note that we do not actually calculate this baseline component because we are using the fixed effect in this equation to identify the student teaching effects. However, assignment of student teachers to mentors may also have a dynamic component which may also lead to bias (Rothstein, 2010). We see, for instance, in Table 1 that teachers appear to be assigned somewhat different students in the year in which they serve as a mentor teacher. We account for observable student characteristics in (1), but unobserved student ability that is correlated with the mentor teacher assignment status would also lead to biased estimates of $\gamma_{3}$_._

We address this possibility in two ways. First, we follow Clotfelter, Ladd, and Vigdor (2006) and Horvath (2015) and estimate models restricted to schools in which students are distributed relatively equitably across classrooms according to prior performance, on the assumption that these schools are also the least likely to nonrandomly sort students to classrooms along unobserved dimensions.^19^ While this data restriction reduces the power of our test, it also limits the possibility of biasing $\gamma_{3}$ by limiting the scope of unobserved sorting of students into (or out of) mentor teacher’s classrooms. Second, we perform a falsification test in which we replace the dependent variable in Equation 4, $Y_{\mathrm{ijst}},$ with students’ *prior* test scores $Y_{is(t-1)},$ and control for twice-lagged test scores $Y_{i(t-2)}\;$ on the right-hand side. If teachers are systematically assigned to “better” classrooms in years they either do or do not host a student teacher—as suggested by the lagged test scores in Table 1—then we would observe an “effect” of student teaching on these lagged test scores in these models attributable to unobserved differences in student background.

Another concern is that Equation 4 makes comparisons between the student teaching year and *all other years* a teacher did not host a student teacher (before and after). However, the literature on student teaching discussed in Section 2, the descriptive trends in Figure 2, and evidence about peer learning in K-12 schools (Jackson & Bruegmann, 2009; Papay et al., 2016; Taylor & Tyler, 2012) suggests that we should treat the years *after* a teacher hosts a student teacher differently than the years before hosting. We therefore extend the model in Equation 4 to include an additional term, *AF_jt_*, indicating whether year *t* is after teacher *j* hosted a student teacher for the first time (and is not itself a student teaching year):

(5) $$Y_{\mathrm{ijst}}=\delta_{0}+\delta_{1}Y_{i(t-1)}+\delta_{2}S_{it}+\delta_{3}{ST}_{jt}+\delta_{4}{AF}_{jt}+\sum_{k}\beta_{k}I_{k}\left(\;\text{Exp}\;\right)+\tau_{js}+\rho_{t}+\varepsilon_{\mathrm{ijst}}$$

The parameters of interest in Equation 5, $\delta_{3}$ and $\delta_{4},$ compare student performance in years during and after student teaching placements (respectively) to student performance in years before the teacher hosted a student teacher, all else equal. Equation 5 amounts to an event study methodology where the comparison group are the years before the event with the possibility of differential impacts after the event.

As noted in Section 2, there is reason to believe that there could be heterogeneous effects of serving as a mentor teacher associated with a mentor teacher’s prior effectiveness as a teacher. For example, if mentor teachers largely hand off instructional responsibility to student teachers, we might see more negative effects in the student teaching year for effective teachers since students are losing instructional time from a more effective teacher. On the other hand, if mentor teachers and student teachers are working in a co-teaching environment, it might be the case that effects of hosting a student teacher are less negative for more effective teachers who may be able to better manage such an arrangement.

To investigate these possibilities, we split the six-year sample into two periods: we use the data from 2009–2010 and 2010–2011 (dropping years in which teachers host a student teacher) to generate an estimate of $\tau_{js};$ and then use the data from 2011–2012 through 2014–2015 to estimate an extension of the model in Equation 5 with additional interactions of mentoring year (ST), after mentoring year (AF) and polynomials of $\tau_{js}.$^20^ Specifically, we use data from 2009–2010 and 2010–2011 to estimate^21^:

(6) $$Y_{\mathrm{ijst}}=\theta_{0}+\theta_{1}Y_{i(t-1)}+\theta_{2}S_{it}+\sum_{k=1}^{50}\beta_{k}I_{k}\left(\;\text{Exp}\;\right)+\tau_{js}+\rho_{t}+\varepsilon_{\mathrm{ijst}}$$

From this equation, we interact the Bayesian-adjusted value added estimate ${\hat{\tau }}_{js},$ which we designate as “Prior VA.” Note, by construction the average teacher in the sample will have ${\hat{\tau }}_{js}$= 0. We then use this measure of Prior VA and data from 2011–2012 through 2014–15 to estimate^22^:

(7) $$Y_{\mathrm{ijst}}=\rho_{0}+\rho_{1}Y_{i\left(t-1\right)}+\rho_{2}S_{it}+\rho_{3}{ST}_{jt}+\rho_{4}{ST}_{jt}\times {\hat{\tau }}_{js}+\rho_{5}{AF}_{jt}+\rho_{6}{AF}_{jt}\times {\hat{\tau }}_{js}+\sum_{k}\beta_{k}I_{k}\left(\;\text{Exp}\;\right)+\rho_{t}\times {\hat{\tau }}_{js}+\tau_{js}+\varepsilon_{\mathrm{ijst}}$$

There are four parameters of interest in Equation 7: $\rho_{3}$ is the average difference in student performance between the student teaching year and years prior to hosting student teacher for teachers with *average* Prior VA; $\rho_{4}$ describes how this relationship changes as Prior VA increases; $\rho_{5}$ represents the average difference in student performance between years *after* hosting a student teacher and years prior to hosting student teacher for teachers with average Prior VA; and $\rho_{6}$ describes how this relationship changes as Prior VA increases. In our preferred specifications of Equation 7, we include both linear and squared terms of Prior VA to capture non-linear relationships between prior mentor teacher effectiveness and student performance during and after student teaching placements.^23^ Importantly, these models also interact Prior VA with the school year to control for regression to the mean (discussed in the next paragraph). It also should be noted that since we use two of our six years of our data to compute Prior VA, models that estimate Equation 7 have significantly fewer observations than those used in Equations 4 and 5.

An important concern with the model in Equation 7 is accounting for measurement error in the Prior VA estimates. We therefore bootstrap the entire two-step procedure (Equations 6 and 7) by first generating a bootstrap sample clustered by teacher to estimate Equation 6, and then estimating Equation 7 for teachers in the bootstrap sample. The standard errors for the coefficients in Equation 7 are based on 1000 bootstrapped samples following this procedure.

**Figure 3.**
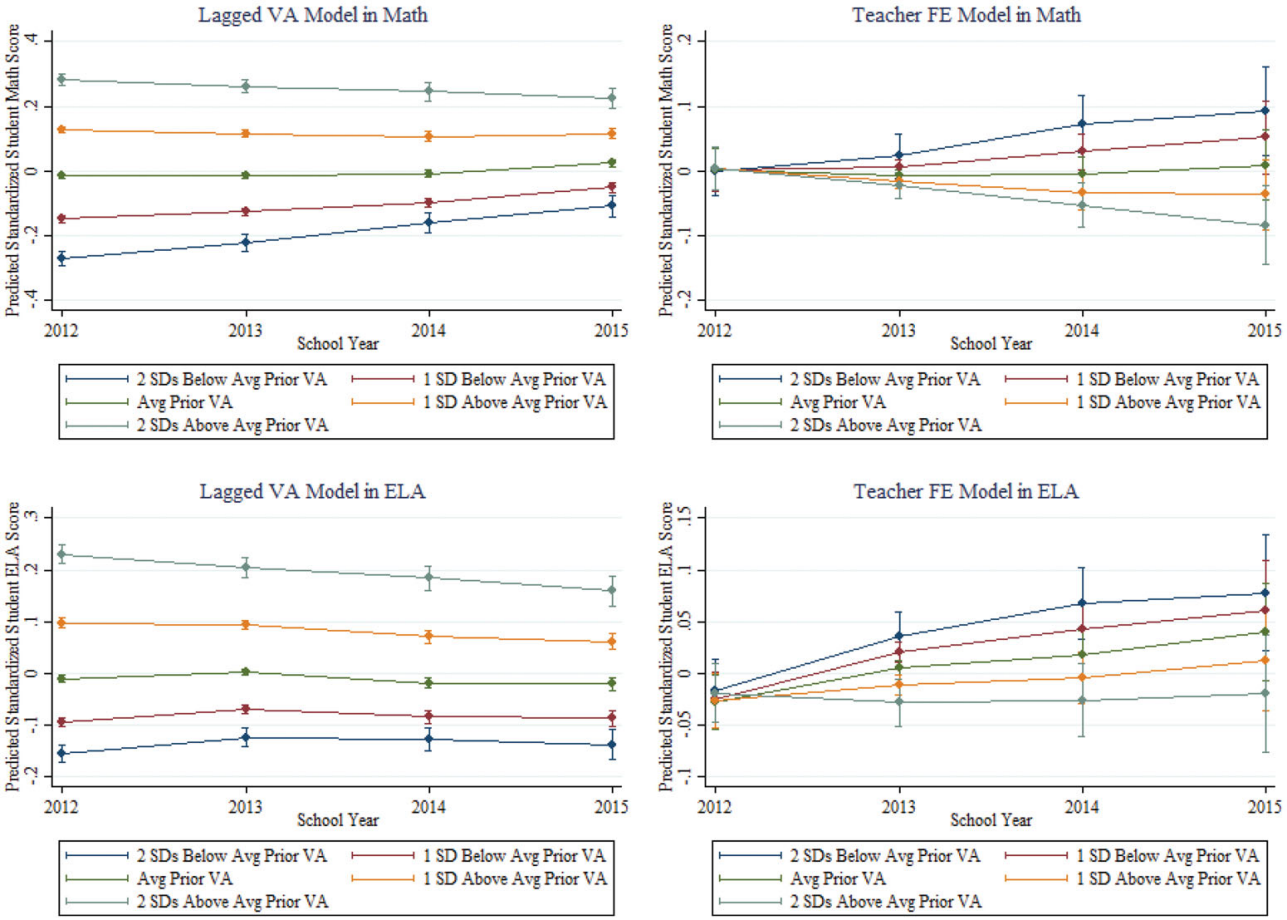
Predicted student achievement by year, prior value added, and model specification. *Note.* ELA = English Language Arts. Predicted student achievement and associated 95% confidence intervals calculated from estimates in Table 3.

Another potential drawback of the model in Equation 7 has to do with its reliance on a mentor teacher’s measure of Prior VA to explain future student learning. As shown by Atteberry, Loeb, and Wyckoff (2015), it is likely that mentor teachers who did particularly well (poorly) in one year, will do worse (better) in subsequent school years; as discussed in Goldhaber and Hansen (2013), this will be true even when value added is shrunken using EB methods (described above), as EB shrinkage is unlikely to provide an accurate estimate of the “permanent component” of teacher performance. This regression to the mean can be seen in Figure 3 which shows the evolution of future value added as a function of measured value added in prior years. From the left panels of this figure (that track predicted value added over time), it is clear that mentor teachers who have high value added in 2009–2010 and 2010–2011, on average, perform closer to the mean in future years, while mentor teachers with low measures of value added in the early period tend to score better. This can also be seen in the right panels of Figure 3, which are derived from a teacher fixed effects models and demonstrates divergence from the baseline year in these models. Given that Equation 6 uses prior value added to explain future performance, the potential regression to the mean likely biases $\rho_{3}$ downwards. We correct for this possibility by interacting prior value added with the time dummies, ρ_t_. Including these terms removes any systematic change in expected value added caused by regression to the mean over time.

A final concern with the model in Equation 7 is that, because Prior VA is collinear with the teacher indicators, we cannot control for the main effect of Prior VA directly in the model. We therefore estimate an alternative to the model in Equation 7 that swaps out the teacher fixed effect for a direct control for prior value added and its square:

(8) $$Y_{\mathrm{ijst}}=\varphi_{0}+\varphi_{1}Y_{i\left(t-1\right)}+\varphi_{2}S_{it}+\varphi_{3}{ST}_{jt}+\;\varphi_{4}{ST}_{jt}\times {\hat{\tau }}_{js}+\varphi_{5}{AF}_{jt}+\varphi_{6}{AF}_{jt}\times {\hat{\tau }}_{js}+\;\varphi_{7}{\hat{\tau }}_{js}+\varphi_{8}{\hat{\tau }}_{js}^{2}+\rho_{t}\times {\hat{\tau }}_{js}+\sum_{k}\beta_{k}I_{k}\left(\;\text{Exp}\;\right)+\varepsilon_{\mathrm{ijst}}$$

As described previously, we estimated standard errors for the coefficients in Equation 8 based on 1000 teacher-clustered bootstrapped samples for the two-step estimation procedure (Equations 6 and 8). While the model in Equation 7 is our preferred specification for investigating heterogeneity by prior value added, we present findings from the models in both Equations 7 and 8 to test the robustness of our findings to these different specifications.

## Results

The estimates of the parameters of interest from Equations 4 and 5 are presented in Table 2. Panel A presents results from math, while Panel B presents results for ELA. Column 1 of Table 2 contains estimates from the model in Equation 4, in which student performance in the student teaching year is compared to student performance in the same teacher’s classroom both before and after the student teaching year. Relative to years without a student teacher, the estimate for math implies that a mentor teacher’s students score 0.018 standard deviations lower in math, all else equal. The comparable estimate for ELA is very close to zero and not statistically significant. Both the math and ELA same-year results represent the net impact of the student teacher’s effectiveness and the student teacher’s impact on the mentor during the student teaching year.

**Table 2.** Effects of hosting a student teacher on standardized student achievement.

	1	2	3	4	5
Panel A: Math					
Year Hosting Student Teacher	−0.018*	−0.004	−0.005	−0.011	0.002
	(0.008)	(0.010)	(0.011)	(0.012)	(0.012)
After Hosting Student Teacher		0.025*	0.022	0.023	0.005
		(0.012)	(0.014)	(0.014)	(0.015)
Students	1,050,090	1,050,090	883,354	853,714	853,714
Sample Years	2010–2015	2010–2015	2010–2015	2010–2015	2010–2015
Sample	Full	Full	Minimal Sorting	Falsification	Falsification
Model	Primary	Primary	Primary	Primary	Falsification
Panel B: ELA					
Year Hosting Student Teacher	0.001	0.013	0.012	0.001	0.001
	(0.007)	(0.009)	(0.011)	(0.011)	(0.010)
After Hosting Student Teacher		0.022*	0.020	0.010	−0.006
		(0.011)	(0.012)	(0.012)	(0.012)
Students	972,201	972,201	773,769	780,206	780,206
Sample Years	2010–2015	2010–2015	2010–2015	2010–2015	2010–2015
Sample	Full	Full	Minimal Sorting	Falsification	Falsification
Model	Primary	Primary	Primary	Primary	Falsification

*Note.* ELA = English Language Arts. All models include a teacher fixed effect, indicators of annual teacher experience and the school year, and also control for the following student control variables interacted by grade: prior performance in math and reading, gender, race/ethnicity, receipt of free or reduced priced lunch, special education status and disability type, Limited English Proficiency indicator, migrant indicator, and homeless indicator. Minimal sorting sample includes only the subset of schools in which there is not significant sorting of students across classrooms by prior performance, and falsification models predict lagged tests scores as a function of twice-lagged test scores and the other variables above. Standard errors clustered at the teacher level are in parentheses. *p* Values from two-sided *t*-test *p < 0.05.

As described by Equation 5, the second column of Table 2 includes *AF*, the identifier for years after a mentor teacher hosts a student teacher. The results with *AF* demonstrate that students perform as well in a mentor teacher’s class when that mentor teacher hosts a student teacher as they would have in prior years. Specifically, when student performance in the student teaching year is compared to the student performance in years before student teaching (the estimated coefficients on “Year Hosting Student Teacher” in column 2 of Table 2), the estimates in both math and ELA are indistinguishable from zero. We therefore conclude that student teaching placements have minimal impact on student performance in the year the mentor teacher hosts a student teacher.

However, the comparisons between years *after* the mentor teacher hosts a student teacher and the years prior to student teaching placement reveal modest, positive effects in both math and ELA. The estimates in each subject suggest that a teacher’s students score 0.02–0.03 standard deviations higher in years after they host a student teacher, all else equal, than in years before the teacher hosts a student teacher.^24^ These estimates control for returns to teacher experience, so cannot be attributed to teachers gaining experience over time. We speculate that these estimates support the notion that mentor teachers benefit from hosting a student teacher and improve their performance in subsequent years. We stress, though, that these effects are modest; for example, the estimated effect in both subjects is less than half of the returns to the first year of teaching experience in that subject (estimated from our models).

The third column of Table 2 restricts the sample to schools that demonstrate minimal sorting between classrooms. This restriction is intended to eliminate observations in which principals can purposely sort students across classrooms, for instance by rewarding teachers with easier-to-educate (along unobserved dimensions) students after serving as a mentor teacher. This type of behavior would mean that the positive, post-hosting effects could be attributed to student composition not controlled for by our student measures rather than improved teaching on the part of mentor teachers. Eliminating the schools where it appears that students are nonrandomly placed in classrooms across years does little to change the coefficients on *AF*, in both Math and ELA the coefficients are statistically similar to those of column 2. However, neither is statistically significant, which we attribute to the larger standard errors caused by restricting the sample size.^25^

The final columns in Table 2 present results from the falsification exercise. In this exercise, Equation 5 is estimated using the test score performance earned by students during the year prior to enrollment in the class taught by the mentor teacher. If teachers are systematically assigned to “better” (in the sense that students have unobserved characteristics associated with better test outcomes) classrooms in years they either do or do not host a student teacher, this would be reflected in the relationships between these years and the prior performance of their students in column 5. The use of twice-lagged test scores reduces the sample some and in order to compare the falsification exercise using the same sample, column 4 reports the model of column 2 using the restricted sample. Column 5 presents the actual falsification exercise and comparing it with column 4 shows that the role of unobservable student characteristics play has minimal, if any, impact on our estimates.

As discussed above, one might expect more or less effective mentors to benefit from a student differentially. Table 3 explores heterogeneity in these effects by the prior value added of mentor teachers, estimated from those years these teachers did not host a student teacher (i.e., Equations 4 and 5). Columns 1 and 5 simply replicate our primary models for the sample of students for whom we can estimate the heterogeneity models, while Columns 2 and 6 present estimates from our preferred mentor teacher fixed effect specification on the full sample. The 3rd and 7th columns repeat this specification on the sample restricted to buildings that do not appear to sort students between classrooms. The final set of columns, the 4th and 8th, present results using the prior value-added models of Equation 7 on the entire sample. The variables of interest are the interaction of prior value added with the binary variables indicating the presence of a student teacher and the identifier for years after hosting a student teacher. In both cases, we include quadratics in prior value added which makes the interpretation of these coefficients difficult.^26^ While it is difficult to interpret quadratic coefficients, none of the interactions of Prior Value Added and student teaching (either concurrent or after) are statistically significant suggesting that if there is heterogeneity in how student teachers impact mentors of different value added, it is too small to detect with this data.

**Table 3.** Effects of hosting a student teacher by prior value added.

	Math				ELA			
	1	2	3	4	5	6	7	8
Year Hosting Student Teacher (ST)	−0.011	−0.003	0.004	0.005	0.011	0.007	0.011	0.013
	(0.015)	(0.025)	(0.031)	(0.062)	(0.016)	(0.025)	(0.031)	(0.018)
After Hosting Student Teacher (After)	0.002	−0.019	−0.015	0.011	0.010	−0.003	0.005	0.018
	(0.019)	(0.034)	(0.044)	(0.028)	(0.020)	(0.034)	(0.041)	(0.022)
Prior VA				0.596***				0.539***
				(0.027)				(0.032)
Prior VA Squared				0.045				0.328***
				(0.062)				(0.107)
Prior VA * ST		0.042	0.085	0.051		−0.027	0.001	−0.046
		(0.117)	(0.123)	(0.084)		(0.158)	(0.180)	(0.105)
Prior VA Squared * ST		−0.017	0.045	−0.022		0.043	0.084	0.044
		(0.153)	(0.153)	(0.121)		(0.190)	(0.213)	(0.150)
Prior VA * After		−0.247	−0.229	−0.127		0.136	−0.103	−0.062
		(0.329)	(0.428)	(0.218)		(0.561)	(0.725)	(0.343)
Prior VA Squared * After		0.247	0.293	0.347		0.642	0.262	0.321
		(0.479)	(0.560)	(0.340)		(0.677)	(0.846)	(0.583)
Students	469,868	469,868	403,110	469,868	420,243	420,243	338,340	420,243
Sample Years	2012–2015	2012–2015	2012–2015	2012–2015	2012–2015	2012–2015	2012–2015	2012–2015
VA Years	2010–2011	2010–2011	2010–2011	2010–2011	2010–2011	2010–2011	2010–2011	2010–2011
Sample	Prior Value Added	Prior Value Added	Minimal Sorting	Prior Value Added	Prior Value Added	Prior Value Added	Minimal Sorting	Prior Value Added
Model	Primary	Primary	Primary	Lagged VA	Primary	Primary	Primary	Lagged VA

*Note.* ELA = English Language Arts; VA = value added. Primary models include a teacher fixed effect, and all models include indicators of annual teacher experience and the school year, interactions between prior VA (linear and squared) and school year, and also control for the following student control variables interacted by grade: prior performance in math and reading, gender, race/ethnicity, receipt of free or reduced priced lunch, special education status and disability type, Limited English Proficiency indicator, migrant indicator, and homeless indicator. Minimal sorting models are estimated only on the subset of schools in which there is not significant sorting of students across classrooms by prior performance. Standard errors clustered at the teacher level (columns 1 and 5) and bootstrapped from 1000 teacher-clustered samples (columns 2–4 and 6–8) are in parentheses. *p* Values from two-sided *t*-test *p < 0.05.

## Discussion and Conclusions

The primary findings we present provide evidence that apprenticeships in grade 4–8 math and ELA classrooms are not generally harmful to the achievement of students in the classrooms in which those apprenticeships take place. This is a somewhat surprising finding given that experienced teachers are, in some cases, turning over their classrooms to truly novice mentees, though perhaps this can be explained by the growing prevalence of “co-teaching” arrangements in the student teaching apprenticeship (e.g., Heck & Bacharach, 2016) or by the collaboration that can occur between a mentor and student teacher. It is also an important finding since schools may be reluctant to host student teachers fearing adverse impacts on student learning, and our null findings help assuage these concerns. Given that this research can only identify the total effects of hosting a student teacher, we leave it to future research to identify specific mechanisms that are driving these effects.

The lack of heterogeneity of the estimated effects by the effectiveness of the mentor teacher is also an important finding. There is currently wide variation in the effectiveness of teachers assigned as a mentor teachers (Goldhaber et al., 2018); a related literature suggests that mentees are better off in the long-run (in terms of their future productivity) when they have a more effective mentor (Goldhaber et al., 2018; Matsko et al., 2018; Ronfeldt et al., 2018), and our findings suggest that there are few short-run costs to the students in the classrooms hosting student teachers. A possible implication of this for policy and practice is that school districts and teacher education programs should have less concern about placing student teachers in classrooms out of fear that these interns harm students. Given that about 3% of teachers host a student teacher in a given year—at least in Washington, the setting of this study (Goldhaber et al., 2018), there is wide scope for school administrators to engage many teachers along these lines. Of course, this suggestion is conditioned by the assumption that those teachers who serve as mentors have similar unobserved characteristics as those who do not serve, something we leave to future research. We also would like to stress that our sample is restricted to tested grades and subjects in late elementary and middle school. It is possible that student teaching outside of these grades, especially the more specialized high school teaching, might generate different results. We therefore caution application of our findings to all student teaching scenarios.

More generally, our findings support a small but growing literature showing that peer learning (Jackson & Bruegmann, 2009; Papay et al., 2016) appears to be an important means of improving incumbent teachers as teachers are found to be more effective *after* having the experience of having served as a mentor. This finding is robust to a number of specification and falsification checks, and has important implications for the schools and districts in which student teaching apprenticeships take place.^27^ In fact, taken together, our findings suggest that school districts can participate in the student teaching process without fear of short-term decreases in student test scores while potentially gaining modest long-term test score increases.

## Data Availability

The research presented here relies on confidential data on K-12 students provided by the Washington Office of Superintendent of Public Instruction through data sharing agreement (DSA) 2015DE-030, and confidential student teaching data provided through individual DSAs with the 15 institutions of higher education participating in the Teacher Education Learning Collaborative. These DSAs all preclude the release of these data to individuals outside the research team, so the data used in this study cannot be made publicly available. All code for the data cleaning and analysis presented in this article are available from the authors upon request.

## Footnotes

1 This figure is based on the number of completers of “traditional” teacher preparation programs, as reported in federal Title II reports (https://title2.ed.gov/Public/DataTools/Tables.aspx). This is a lower bound on the total number of preservice teaching internships because many alternative programs (e.g., Teach for America) require some form of preservice teaching apprenticeship prior to workforce entry.

2 There is some qualitative evidence suggesting that student teaching benefits K-12 students (e.g., Field & Philpott, 2000; Kerry & Shelton Mayes, 1995).

3 For instance, only 20% of states required that a mentor teacher hold a minimum level of professional experience or demonstrate mentoring skills in 2011 (Greenberg et al., 2011).

4 Similarly, locally negotiated memorandums of understanding between TEPs and school systems tend to focus on the broad nature of the student teaching experience without getting into the specifics of whether internships ought to take place in specific types of schools or be overseen by specific types of mentor teachers (Goldhaber, Krieg, & Theobald, 2014).

5 There is little quantitative evidence in support or opposition to this hypothesis for student teaching, but some evidence suggesting that the matching of higher and lower performing inservice teachers benefits both (Papay et al., 2016).

6 p. 127.

7 Specifically, Jackson and Bruegmann (2009) find that teachers tend to be more effective when they work in schools with more effective peers, while Papay et al. (2016) find large test score gains associated with the pairing of low-performing and high-performing teachers for professional development, with the gains concentrated in the classrooms of the low-performing teachers.

8 One exception is Bacharach, Heck, and Dahlberg (2010), who find some evidence that students taught by a student teacher in a co-teaching setting have greater learning gains than students taught by a student teacher in a traditional setting.

9 The institutions participating in TELC and that provided data for this study include: Central Washington University, City University, Evergreen State College, Gonzaga University, Northwest University, Pacific Lutheran University, St. Martin’s University, Seattle Pacific University, Seattle University, University of Washington Bothell, University of Washington Seattle, University of Washington Tacoma, Washington State University, Western Governors University, and Western Washington University. The 6 institutions that are not participating in TELC include one relatively (for Washington) large public institution in terms of teacher supply, Eastern Washington University, and five smaller private institutions: Antioch University, Heritage University, University of Puget Sound, Walla Walla University, and Whitworth University. Only two of these, the University of Puget Sound and Antioch University, are west of the Cascades.

10 13 TEPs provided data for all six of these years, while 2 TEPs (both small private institutions) provided data for only three of these six years. Although programs provided data on mentor teachers in a variety of formats, we are able to match 97% of teacher candidates in the TELC data whose program provided mentor teaching information and who did their student teaching in public schools in Washington to a valid mentor teacher observation in the OSPI data.

11 CEDARS data includes fields designed to link students to their individual teachers, based on reported schedules. However, limitations of reporting standards and practices across the state may result in ambiguities or inaccuracies around these links.

12 Note that, while many placements occurred in private schools and out-of-state schools, we do not consider these placements in this analysis because we do not have data about these schools or the students and teachers in these schools.

13 We can obtain this estimate because the OSPI data include information the institutions from which teachers (not teacher candidates) receive their teaching credentials. About 22 percent of new teachers come in from out of state (and receive an OSPI credential) (Goldhaber, Liddle, & Theobald, 2013), and are not included in these figures or this analysis.

14 The gap in coverage of student teachers is primarily driven by the fact that the three largest TEPs not participating in TELC are all in the eastern half of the state.

15 Our empirical models rely upon within-mentor variation in student test scores. Mis-identifying a mentor as not hosting a student teacher when in fact they do simply results in that mentor being dropped from the analysis (since there is no variation in their hosting status over the time observed). This would result in biasing our empirical models only to the extent that student teachers from the one west-side program not observed in the TELC data have a differential effect on their mentor teachers’ classroom relative to our observed mentors, a possibility we consider remote.

16 Appendix Table A1 compares candidate information (including average licensure test scores on the Washington Educator Skills Test – Basic [WEST-B] in math, reading, and writing) for student teachers who are and are not in this analytic sample. Appendix Table A1 also illustrates the prevalence of departmentalized instruction (i.e., teachers teaching only math or ELA) and self-contained instruction (i.e., teachers teaching both math and ELA) in the sample.

17 For brevity, we do not show ELA descriptive statistics because they are qualitatively similar to the math descriptive statistics shown in Table 1. The ELA descriptive statistics are in Appendix Table A2.

18 The experience and year effects in this model can be separately identified because of teachers who have gaps in their teaching experience. However, this raises the possibility that this source of identification may lead to imprecise controls for returns to teacher experience. We therefore experiment with models that omit the year controls and find that the results are nearly identical, likely because (as discussed for Figure 2) the relationship between experience and value added is relatively weak for the more experienced teachers who tend to host student teachers.

19 Both approaches are intended to create “apparently random samples” by dropping students and teachers in schools that display considerable tracking of students to classroom along observed dimensions. We follow the Horvath (2015) methodology of dropping all schools in which an F-test rejects the null hypothesis that classrooms within schools do not predict student prior performance.

20 We chose these samples to maximize the number of teachers who have an estimate of prior value added from the first period and host a student teacher in the second period.

21 Note that this model is different than the model in Equation 1 because value added is pooled across 2009–2010 and 2010–2011 rather than across 2009–2010 through 2014–2015. This raises the potential concern that, if relationships btween covariates in the model are different in this earlier period than across the entire sample, it could bias our findings. We therefore estimate models on the full sample that allow these relationships to vary for the two time periods, and compare them to estimates from models that do not. The resulting correlation is 0.9991, so we are confident that this alternative specification generates substantially the same results as those of the earlier equations.

22 Empirical Bayes (EB) methods shrink the value added estimates back to the grand mean of the value-added distribution in proportion to the standard error of each estimate. EB shrinkage does not account for the uncertainty in the grand mean, suggesting that estimates may shrink too much under this procedure (McCaffrey, Sass, Lockwood, & Mihaly, 2009); this approach, however, ensures that estimates in the tail of the distribution are not disproportionately estimated with large standard errors. And as discussed in Jacob and Lefgren (2008), shrinking value-added estimates before including them as predictors removes bias due to measurement error and uncertainty in these estimates.

23 We experiment with higher order polynomials of prior value added and selected the quadratic based upon highest F-test of the coefficients on these polynomial models.

24 We refer to these effects as modest because they are roughly half the estimated effect of a one standard increase in average peer quality (Jackson & Bruegmann, 2009) and less than a fourth of the estimated effect of assignment of a low-performing teacher to work with a higher-performing peer in the school (Papay et al., 2016).

25 Note the decrease in the sample size by 15% increases the standard errors by 9%, which is nearly exactly the growth in the standard errors between columns 2 and 3 of Table 2.

26 We explored higher order polynomials which generally produced similar results to the quadratic. The one case where there was a difference was that math students of the lowest performing mentors did statistically significantly worse in the lagged models. We chose not to report these results and focus on the more conservative quadratic estimates.

27 As one example, given that teachers report that serving as a mentor is time consuming and there is little financial reward—by one estimate, the average compensation that mentor teachers receive in 2012–2013 is $232, far lower than the nearly $1600 (adjusted for inflation) that was typical back in 1959 (Fives, Mills, & Dacey, 2016)—there is a compelling argument for additional rewards for serving in this role.

## Appendix Table A1. Candidate summary statistics (Student teaching between 2010 and 2015, West of Cascades)

**Table:**

	Not in Sample	In Sample	Math Only	ELA Only	Math and ELA
Panel A: Candidate Institution					
City University	8.0%	12.5%	19.2%	8.9%	11.8%
Central Washington University	13.0%	10.9%	13.6%	3.4%	12.6%
Evergreen State College	2.4%	3.1%	4.8%	4.6%	2.0%
Gonzaga University	0%	0.0%	0.0%	0.0%	0.0%
Northwest University	2.5%	3.4%	2.6%	7.2%	2.4%
Pacific Lutheran University	8.0%	5.6%	4.2%	7.2%	5.5%
St. Martin’s University	3.9%	4.0%	8.1%	5.7%	2.2%
Seattle Pacific University	10.4%	7.1%	8.4%	8.0%	6.4%
Seattle University	1.2%	0.4%	0.3%	0.3%	0.5%
University of Washington Bothell	3.4%	2.8%	1.0%	1.7%	3.7%
University of Washington Seattle	9.5%	9.6%	3.3%	14.4%	10.0%
University of Washington Tacoma	3.6%	5.4%	9.1%	5.5%	4.3%
Western Governors University	3.1%	3.0%	5.8%	0.0%	3.2%
Washington State University	10.1%	12.0%	5.8%	9.8%	13.7%
Western Washington University	21.0%	20.1%	10.7%	23.2%	21.8%
Unique candidates	5,870	1,699	308	348	1,043
Panel B: Candidate WEST-B Scores					
WEST-B Math	279.4	280.9	286.0	278.5	280.2
	(17.5)	(16.4)	(15.4)	(17.3)	(16.1)
WEST-B Reading	272.2	273.2	274.9	276.2	271.7
	(15.1)	(14.5)	(13.6)	(13.2)	(14.9)
WEST-B Writing	265.1	266.0	265.8	270.5	264.6
	(18.1)	(18.4)	(18.6)	(16.3)	(18.8)
Unique candidates	5,474	1,589	290	322	977

*Note.* ELA = English Language Arts. Summary statistics limited to candidates in TELC dataset who student taught west of the Cascades between 2009–2010 and 2014–2015.

## Appendix Table A2. Student summary statistics (Mentor teachers in ELA only)

**Table:**

	All	Before student teaching year	Student teaching year	After student teaching year
Prior score in math (standardized)	0.076	0.081	0.057	0.087
	(0.988)	(0.988)	(0.996)	(0.981)
Prior score in ELA (standardized)	0.084	0.085	0.071	0.093
	(0.957)	(0.955)	(0.965)	(0.954)
Female	0.496	0.494	0.496	0.496
American Indian	0.012	0.013	0.012	0.010*
Asian/Pacific Isl.	0.125	0.118	0.139**	0.123
Black	0.065	0.058	0.078***	0.061
Hispanic	0.143	0.133	0.148**	0.150**
White	0.592	0.622	0.565***	0.584***
Learning disability	0.053	0.053	0.055	0.050
Special Education	0.112	0.112	0.116	0.110
Gifted	0.079	0.074	0.075	0.086
Limited English	0.053	0.045	0.058***	0.057**
Free/Reduced Lunch	0.415	0.399	0.436**	0.414
Unique teachers	1,128	1,128	1,128	1,128
Unique student teachers	1,392	0	1,392	0
Unique students	152,022	53,780	42,600	55,642

*Note.* ELA = English Language Arts. Summary statistics limited to teachers who hosted at least one student teacher in a grade 4–8 ELA classroom between 2009–2010 and 2014–2015. *p* Values from two-sided *t*-test: **p*< 0.05; ***p* < 0.01; ****p* < 0.001.

## References

[CIT0001] Anderson, L. M., & Stillman, J. A. (2013). Student teaching’s contribution to preservice teacher development: A review of research focused on the preparation of teachers for urban and high-needs contexts. *Review of Educational Research*, 83(1), 3–69. doi:10.3102/0034654312468619

[CIT0002] Atteberry, A., Loeb, S., & Wyckoff, J. (2015). Do first impressions matter? Predicting early career teacher effectiveness. *AERA Open*, 1(4). doi:10.1177/2332858415607834

[CIT0003] Bacharach, N., Heck, T. W., & Dahlberg, K. (2010). Changing the face of student teaching through coteaching. *Action in Teacher Education*, 32(1), 3–14. doi:10.1080/01626620.2010.10463538

[CIT0004] Borko, H., & Mayfield, V. (1995). The roles of cooperating teacher and university supervisor in learning to teach. *Teaching & Teacher Education*, 11(5), 501–518. doi:10.1016/0742-051X(95)00008-8

[CIT0007] Clarke, A., Triggs, V., & Nielsen, W. (2014). Cooperating teacher participation in teacher education: A review of the literature. *Review of Educational Research*, 84(2), 163–202. doi:10.3102/0034654313499618

[CIT0008] Clotfelter, C. T., Ladd, H. F., & Vigdor, J. L. (2006). Teacher-student matching and the assessment of teacher effectiveness. *Journal of Human Resources*, 41, 778–820.

[CIT0009] Field, K., & Philpott, C. (2000). The impact of hosting student teachers on school effectiveness and school improvement. *Journal of In-Service Education*, 26(1), 115–137. doi:10.1080/13674580000200107

[CIT0010] Fives, H., Mills, T. M., & Dacey, C. M. (2016). Cooperating teacher compensation and benefits: Comparing 1957–1958 and 2012–2013. *Journal of Teacher Education*, 67(2), 105–119. doi:10.1177/0022487115626428

[CIT0011] Ganser, T. (2002). How teachers compare the roles of cooperating teacher and mentor. *In the Educational Forum*, 66(4), 380–385. doi:10.1080/00131720208984858

[CIT0037] Goldhaber, D., & Hansen, M. (2013). Is it just a bad class? Assessing the long-term stability of estimated teacher performance. *Economica*, 80(319), 589–612. doi:10.1111/ecca.12002

[CIT0012] Goldhaber, D., Krieg, J., & Theobald, R. (2014). Knocking on the door to the teaching profession? Modeling the entry of prospective teachers into the workforce. *Economics of Education Review*, 43, 106–124. doi:10.1016/j.econedurev.2014.10.003

[CIT0013] Goldhaber, D., Krieg, J. M., & Theobald, R. (2017). Does the match matter? Exploring whether student teaching experiences affect teacher effectiveness. *American Educational Research Journal*, 54(2), 325–359. doi:10.3102/0002831217690516

[CIT0014] Goldhaber, D., Krieg, J., & Theobald, R. (2018). *Effective like me? Does having a more productive mentor improve the productivity of mentees?* (National Center for Analysis of Longitudinal Data in Education Research Working Paper No. 208-1118-1). Washington, DC.

[CIT0015] Goldhaber, D., Liddle, S., & Theobald, R. (2013). The gateway to the profession: Evaluating teacher preparation programs based on student achievement. *Economics of Education Review*, 34, 29–44. doi:10.1016/j.econedurev.2013.01.011

[CIT0016] Graham, B. (2006). Conditions for successful field experiences: Perceptions of mentor teachers. *Teaching and Teacher Education*, 22(8), 1118–1129. doi:10.1016/j.tate.2006.07.007

[CIT0017] Greenberg, J., Pomerance, L., & Walsh, K. (2011). *Student teaching in the United States*. Washington, DC: National Council on Teacher Quality.

[CIT0020] Heck, T. W., & Bacharach, N. (2016). A better model for student teaching. *Educational Leadership*, 73(4), 24–29.

[CIT0021] Hoffman, J. V., Wetzel, M. M., Maloch, B., Greeter, E., Taylor, L., DeJulio, S., & Vlach, S. K. (2015). What can we learn from studying the coaching interactions between mentor teachers and preservice teachers? A literature review. *Teaching and Teacher Education*, 52, 99–112. doi:10.1016/j.tate.2015.09.004

[CIT0039] Horvath, H. (2015). Classroom assignment policies and implications for teacher value-added estimation. Retrieved from http://conference.iza.org\conference_files\ESSLE2015

[CIT0022] Hurd, S. (2007). *The impact of trainee teachers on school achievement: A review of research*. Milton Keynes: Centre for Research & Development in Teacher Education, The Open University.

[CIT0041] Jackson, C. K., & Bruegmann, E. (2009). Teaching students and teaching each other: The importance of peer learning for teachers. *American Economic Journal: Applied Economics*, 1(4), 85–108. doi:10.1257/app.1.4.85

[CIT0023] Jacob, B. A., & Lefgren, L. (2008). Can principals identify effective teachers? Evidence on subjective performance evaluation in education. *Journal of Labor Economics*, 26(1), 101–136. doi:10.1086/522974

[CIT0024] Kerry, T., & Shelton Mayes, A. (Eds). (1995). *Issues in mentoring*. Buckingham, UK: Open University Press.

[CIT0025] Krieg, J. M., Theobald, R., & Goldhaber, D. (2016). A foot in the door: Exploring the role of student teaching assignments in teachers’ initial job placements. *Educational Evaluation and Policy Analysis*, 38(2), 364–388. doi:10.3102/0162373716630739

[CIT0026] Krieg, J., Goldhaber, D., & Theobald, R. (in press). Teacher candidate apprenticeships: Assessing the who and where of student teaching. *Journal of Teacher Education*, doi:10.1177/0022487119858983

[CIT0043] Ladd, H. F., & Sorensen, L. C. (2017). Returns to teacher experience: Student achievement and motivation in middle school. *Education Finance and Policy*, 12(2), 241–279. doi:10.1162/EDFP_a_00194

[CIT0045] Matsko, K. K., Ronfeldt, M., Nolan, H. G., Klugman, J., Reninger, M., & Brockman, S. (2018). Cooperating teacher as model and coach: What leads to student teachers' perceptions of preparedness? *Journal of Teacher Education*, 71(1), 41–62. doi:10.1177/0022487118791992

[CIT0029] McCaffrey, D. F., Sass, T. R., Lockwood, J. R., & Mihaly, K. (2009). The intertemporal variability of teacher effect estimates. *Education Finance and Policy*, 4(4), 572–606. doi:10.1162/edfp.2009.4.4.572

[CIT0046] National Council for Accreditation of Teacher Education. (2010). Transforming teacher education through clinical practice: A national strategy to prepare effective teachers. Report of the Blue Ribbon Pane on Clinical Preparaton for Improved Student Learning. Retrieved from https://eric.ed.gov/?id=ED512807

[CIT0048] Papay, J., Taylor, E., Tyler, J., & Laski, M. (2016). Learning job skills from colleagues at work: Evidence from a afield experiment using teacher performance data (NBER Working Paper 21986). doi:10.3386/w21986

[CIT0050] Rivkin, S. G., Hanushek, E. A., & Kain, J. F. (2005). Teachers, schools, and academic achievement. *Econometrica*, 73(2), 417–458. doi:10.1111/j.1468-0262.2005.00584.x

[CIT0052] Rockoff, J. (2004). The impact of individual teachers on student achievement: Evidence from panel data. *American Economic Review*, 94(2), 247–252. doi:10.1257/0002828041302244

[CIT0031] Ronfeldt, M. (2012). Where should student teachers learn to teach? Effects of field placement school characteristics on teacher retention and effectiveness. *Educational Evaluation and Policy Analysis*, 34(1), 3–26. doi:10.3102/0162373711420865

[CIT0032] Ronfeldt, M. (2015). Field placement schools and instructional effectiveness. *Journal of Teacher Education*, 66(4), 304–320.

[CIT0033] Ronfeldt, M., Brockman, S., & Campbell, S. (2018). Does cooperating teachers’ instructional effectiveness improve preservice teachers’ future performance? *Educational Researcher*, 47(7), 405–418.

[CIT0054] Rothstein, J. (2010). Teacher quality in educational production: Tracking, decay, and student achievement. *The Quarterly Journal of Economics*, 125(1), 175–214. doi:10.1162/qjec.2010.125.1.175

[CIT0034] St. John, E., Goldhaber, D., Krieg, J., & Theobald, R. (2018). *How the match gets made: Exploring student teacher placements across teacher education programs, districts, and schools* (CALDER Working Paper 111018). Washington, DC: National Center for Analysis of Longitudinal Data in Education Research.

[CIT0035] Taylor, E. S., & Tyler, J. H. (2012). The effect of evaluation on teacher performance. *American Economic Review*, 102(7), 3628–3651. doi:10.1257/aer.102.7.3628

[CIT0036] Zeichner, K. M. (2009). *Teacher education and the struggle for social justice*. New York, NY: Routledge.

